# Adiposity in Early, Middle and Later Adult Life and Cardiometabolic Risk Markers in Later Life; Findings from the British Regional Heart Study

**DOI:** 10.1371/journal.pone.0114289

**Published:** 2014-12-04

**Authors:** Venediktos V. Kapetanakis, Alicja R. Rudnicka, Andrea K. Wathern, Lucy Lennon, Olia Papacosta, Derek G. Cook, S. Goya Wannamethee, Peter H. Whincup, Christopher G. Owen

**Affiliations:** 1 Population Health Research Institute, St George’s, University of London, Cranmer Terrace, London, United Kingdom; 2 Primary Care and Population Health, University College London Medical School, Royal Free Campus, London, United Kingdom; Stellenbosch University, South Africa

## Abstract

***Objectives:*** This research investigates the associations between body mass index (BMI) at 21, 40–59, 60–79 years of age on cardiometabolic risk markers at 60–79 years.

***Methods:*** A prospective study of 3464 British men with BMI measured at 40–59 and 60–79 years, when cardiometabolic risk was assessed. BMI at 21 years was ascertained from military records, or recalled from middle-age (adjusted for reporting bias); associations between BMI at different ages and later cardiometabolic risk markers were examined using linear regression. Sensitive period, accumulation and mobility life course models were devised for high BMI (defined as BMI≥75^th^ centile) and compared with a saturated BMI trajectory model.

***Results:*** At ages 21, 40–59 and 60–79 years, prevalences of overweight (BMI≥25 kg/m^2^) were 12%, 53%, 70%, and obesity (≥30 kg/m^2^) 1.6%, 6.6%, and 17.6%, respectively. BMI at 21 years was positively associated with serum insulin, blood glucose, and HbA1c at 60–79 years, with increases of 1.5% (95%CI 0.8,2.3%), 0.4% (0.1,0.6%), 0.3% (0.1,0.4%) per 1 kg/m^2^, respectively, but showed no associations with blood pressure or blood cholesterol. However, these associations were modest compared to those between BMI at 60–79 years and serum insulin, blood glucose and HbA1c at 60–79 years, with increases of 8.6% (8.0,9.2%), 0.7% (0.5,0.9%), and 0.5% (0.4,0.7%) per 1 kg/m^2^, respectively. BMI at 60–79 years was also associated with total cholesterol and blood pressure. Associations for BMI at 40–59 years were mainly consistent with those of BMI at 60–79 years. None of the life course models fitted the data as well as the saturated model for serum insulin. A sensitive period at 50 years for glucose and HbA1c and sensitive period at 70 years for blood pressure were identified.

***Conclusions:*** In this cohort of men who were thin compared to more contemporary cohorts, BMI in later life was the dominant influence on cardiovascular and diabetes risk. BMI in early adult life may have a small long-term effect on diabetes risk.

## Introduction

Adult overweight and obesity are leading global risk factors for mortality and disability, particularly in middle and high income countries.[1] Prevalences of overweight and obesity in middle-aged and older adults have increased markedly across the world in recent decades;[2]–[4] and are strongly associated with major chronic diseases, including cardiovascular disease and type 2 diabetes.[5]–[8] However, there is growing concern about the emergence of overweight and obesity earlier in the life course, during early adult life, adolescence and in childhood. The early development of adiposity has immediate adverse effects on established cardiovascular risk factors [9] and diabetic precursors,[10], [11] which may persist,[12] as well as strongly predisposing to overweight and obesity in middle-age.[13], [14] It is possible that higher levels of adiposity for increasing duration may have serious additional adverse long-term health consequences, particularly for cardiovascular disease and type 2 diabetes in middle and later life.[15], [16] However, its effects have not been fully elucidated.

Further elucidation of the impact of adiposity in early adult life on cardiovascular disease and diabetes requires data from long-term cohort studies with accurate information on adiposity in early adult life, middle-age and later adult life as well as long-term prospective data on cardiovascular and diabetes risk.[16], [17] Few prospective studies (either birth cohort studies or conventional cohorts from adult life) have data of this kind.[5], [16], [18]–[20] We have obtained data from military service records on measured weight and height in early adult life for participants in an established cohort of British men examined at 40–59 and 60–79 years in order to study the impact of adiposity in early, middle and later adult life on cardiometabolic risk at 60–79 years. Throughout this report body mass index (BMI) in early adult (mean age 21 years), middle-age (mean age 50 years) and late adulthood (mean age 70 years) are referred to as BMI-21, BMI-50 and BMI-70 respectively.

## Methods

The British Regional Heart Study (BRHS) is a long-term prospective study of risk factors for cardiovascular disease and type 2 diabetes among middle-aged and older British men. It is based on 7735 men (78% response) born between 1919 and 1939 who were recruited in 1978–80 aged 40–59 years from a single General Practice in each of 24 British towns.[21] The BRHS was a predominantly white European cohort of men (>99% white European). Ethical approval for the BRHS including the investigation of military records reported here has been provided by the London Multicentre Research Ethics Committee and its successor, the Research Ethics Committee for London Central (approval reference MREC/02/02/91). Study participants had a detailed examination and questionnaire assessment at entry and completed periodic postal questionnaires about their health in 1983–5, 1992 and 1996; in 1996 they were asked to recall their weight at age 21 years. In 1998–2000, all surviving men (n = 5516) were invited for a detailed 20 year follow-up examination aged 60–79 years, of whom 4252 men (77%) attended for re-examination. In 2007, a questionnaire was sent to all surviving men enquiring about military service in early adulthood, either during World War II (1939–1945) or during National Service (1945–1963), including details of service period and service number where available. Participants were asked for written permission to allow the researchers to obtain relevant health information from the military records.

### Baseline and 20 year assessment

At both assessments, men provided information on medical history, use of regular medications and lifestyle behaviour (including smoking status, physical activity, alcohol intake and social class). Standing height and weight were measured with participants in light clothing without shoes; height was measured to the last complete 0.1 cm using a Harpenden stadiometer, and weight to the last 0.1 kg using regularly calibrated scales. At the 20 year assessment two resting measurements of seated blood pressure were measured in the right arm using an automated blood pressure monitor (Dinamap 1846, Critikon Corporation, Tampa, FL, USA) with an appropriately sized cuff; values were adjusted for overreading of systolic blood pressure by the instrument [22] and for observer variation.[23] A fasting venous blood sample was collected from all participating men. Details of analytic methods have been previously documented.[24] HbA1c was measured in whole blood using high performance liquid chromatography. Serum total cholesterol and HDL-cholesterol were measured using an auto-analyser (Hitachi 747, Hitachi, Tokyo, Japan). LDL-cholesterol was calculated using the Friedrickson–Friedwald equation.[25] Plasma glucose was measured using a glucose oxidase method; serum insulin was measured using an ELISA method that does not cross-react with proinsulin.[26] All cardiovascular risk factors and metabolic markers were adjusted for time of day, and fasting duration.[24].

### Weight and height during military service

With ethical committee approval, military records were sought for all study participants (including deceased participants) except those who refused consent or confirmed that they had not undertaken military service. All information obtained from military records was de-identified prior to analysis, being linked only on the basis of the unique study identifier. Military service records (electronically indexed by name, date or birth, and service number) were retrieved from the single UK repository (TNT Ltd, Swadlincote, Derbyshire) and reviewed by a trained researcher. All measures of weight and height recorded at entry to the armed services and at other times during service were recorded

### Data management and statistical methods

Weight at 21 was obtained using armed service records or, where not available, from self-reported weight at age 21 recalled in 1996. For armed service records, weight recorded between 20 and 22 years, using the value recorded closest to 21 years, was used as a measurement of weight at 21 years without adjustments. Where measurements between 17–19 or 23–25 years (but not 20–22 years) were available, weight at 21 years was estimated from a multi-level model fitted using all weight measurements recorded between 17 and 25 years of all individual participants, adjusting for age (allowing for a quadratic relationship) and period of enlistment (1934–38, 1939–45, 1946–50, 1951–55, 1956–75). The model included a random intercept and random slope for the linear term of age to take account of clustering of measures within individual. When recalled weight in 1996 was used, this was adjusted for recall bias quantified by comparisons of measured (within the age range 20–22 years and closest to age 21) and recalled weight at 21 years in 694 individuals with data from both sources. Recall was associated with a small overestimation of weight at 21 years in men who were thinner than average (mean estimated bias across the range of measured weight was 1.5 kg with a maximum of 6.9 kg estimated at the lowest limit of the range of measured weight) (Figure S1 in File S1). Height measurements recorded in military records were used to provide an estimate of height at 21 years using similar methods as for weight. Shrinkage between 21 years and measured height at mean 50 years (was 0.2 cm; 95%CI 0 cm to 0.3 cm) was modelled using data from 1248 individuals with height from both sources. Body mass index (BMI) at mean 21, 50 and 70 years was calculated as weight/height squared in kg/m^2^.

### Statistical methods

Physical and metabolic factors were summarised by quintiles of BMI-21 to examine patterns of association. Linear regression was used to assess the associations between BMI-21, BMI-50, and BMI-70 and cardiovascular and metabolic risk markers in late adulthood (adjusting for age at outcome and town as a fixed effect); percentage changes were examined for log transformed markers. The associations between BMI-70 and cardiometabolic markers were stratified by quintiles of BMI-21 to examine effect modification using a test for trend obtained from meta-regression. Patterns of high BMI over the life course were defined according to whether an individual was below (0) or on/above (1) the 75th percentile of the BMI distribution at each age period (early, middle, later adult life) and were denoted by a triplet of zeros and ones (8 trajectories in all). The regression coefficients for all trajectories were used to examine life course patterning of cardiometabolic risk, adjusting for age at outcome and town (saturated model). Other life course models including sensitive period, accumulation and exposure change models [27] were also investigated. Sensitive period models assume that cardiometabolic risk markers may depend on some or all the periods in which participants have had high BMI, but having high BMI during a particular age period is associated with a greater effect.[27] Under the hypothesis of accumulation, having high BMI has a cumulative effect to cardiovascular risk markers in late adulthood irrespectively of the time one has had high BMI.[27] The assumption behind an exposure change model is that any upward change in BMI classification (from no high BMI to high BMI), and any downward change in BMI classification (from high BMI to no high BMI) have equal effect irrespectively of the age period that this change has happened (from early to middle adulthood, or from middle to late adulthood). These models are special cases of the saturated model that includes all 8 trajectories of high BMI over the life course. The saturated model was compared with the simpler models using likelihood ratio tests to examine whether the simpler model fitted the data equally well.[27].

## Results

Among 4252 men who attended for examination at both mean 50 and 70 years (78% and 77% response rate among survivors respectively), information on weight at 21 years was available for 3464 (82%) men. Among these, 1252 men had data available from military records and 2906 men had participant recall from the 1996 questionnaire; 694 men had data from both sources.

**Figure 1:**
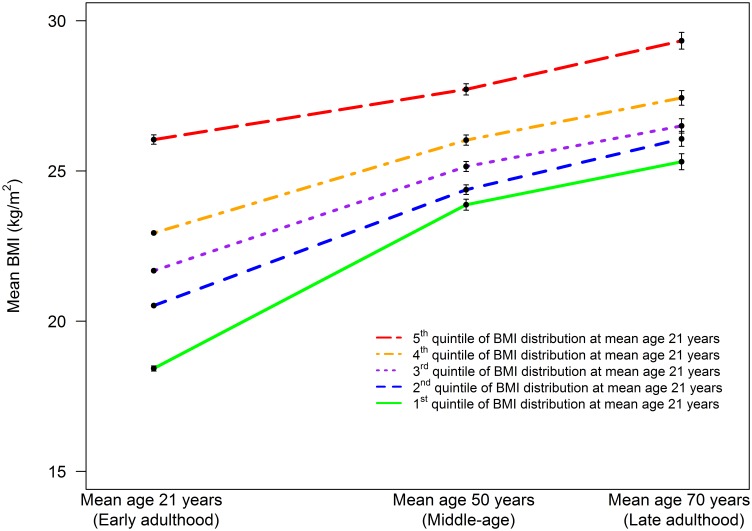
Distribution of BMI at mean age 21 years (early adulthood), 50 years (middle-age), and 70 years (late adulthood).

**Figure 2:**
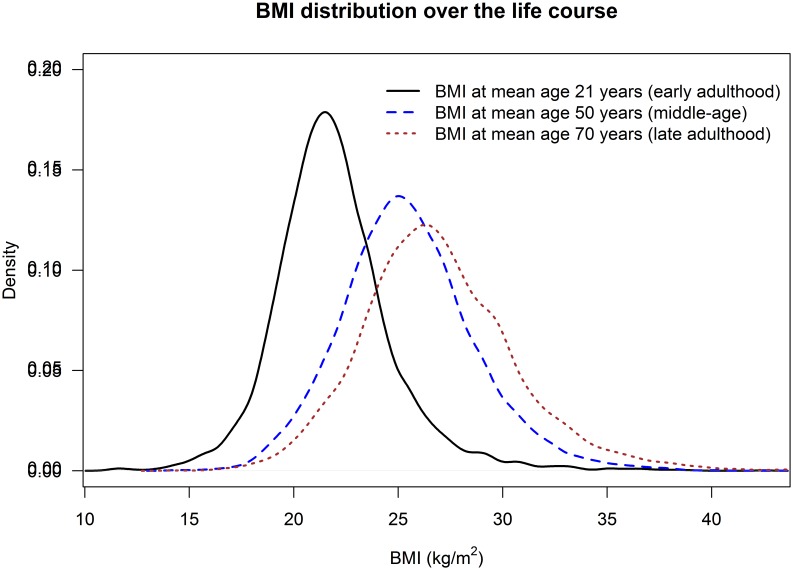
Mean BMI at mean age 21, 50 and 70 years, stratified by quintile of BMI distribution at age 21 (quintiles formed by using all available data at age 21; lines from bottom to top correspond to 1^st^, 2^nd^, 3^rd^, 4^th^, and 5^th^ quintiles, respectively). Note: Individuals may be moving across quintiles.

Table 1 presents the cohort characteristics at mean age 21, 50 and 70 years by quintile of BMI-21 in all men. Mean BMI rose between early, middle and late adulthood, whereas height was reduced marginally between age 21 and 50 years, with a small shrinkage (1.5 cm, p<0.001) between mean age 50 and 70 years. Figure 1 illustrates the distribution of BMI-21, BMI-50 and BMI-70. Both prevalences of overweight (defined as BMI ≥25 kg/m^2^) and obesity (defined as BMI ≥30 kg/m^2^) rose steeply with increasing age (12%, 53%, 70%; 1.6%, 6.6%, 17.6% respectively). A comparison of BMI-21, BMI-50 and BMI-70 between individuals with available data at all age periods and those excluded from our analyses because of missing values in at least one age period showed small differences between the two groups at mean age 21 and 50 years (difference −0.18 kg/m2, p = 0.04; 0.17 kg/m2, p = 0.019; respectively), and little difference in BMI-70 (-0.07 kg/m2, p = 0.62). Figure 2 shows the trajectory of early BMI quintiles into middle-age and later life. The absolute and relative rates of increase in average BMI between early and middle-age were highest for those in the lowest quintile of BMI-21 and lowest for those in the highest quintile. While trajectories of average BMI did not cross over the life course, only 18% of men remained in the same quintile of BMI distribution at all age periods. For example, treating individuals in each quintile of BMI at mean age 21 years as five separate groups, those in the bottom quintile had the lowest average BMI at both mean age 50 and 70 years. However, only 40% and 38% of individuals in the bottom quintile of BMI at mean age 21 years were amongst those in the lowest BMI quintile at mean age 50 and at mean age 70 years, respectively.

**Table 1:** Cohort characteristics in early, middle, and late adulthood in all men, and by quintile of BMI in early adulthood.

Characteristic			Percentile group of BMI at age 21 years									
	All		0%–20%		21%–40%		41%–60%		61%–80%		81%–100%	
	n	Mean ± SD/%	n	Mean ± SD	n	Mean ± SD	n	Mean ± SD	n	Mean ± SD	n	Mean ± SD
**Measurements at mean age 21** **years (early adulthood)**												
BMI (kg/m^2^)	3464	22.0±3.0	703	18.4±1.4	671	20.5±0.4	680	21.7±0.3	671	22.9±0.4	739	26.1±2.6
normal-weight (BMI<25 kg/m^2^)	3043	87.9%										
overweight (25 kg/m^2^≤BMI<30 kg/m^2^)	367	10.6%										
obese (BMI≥30 kg/m^2^)	54	1.6%										
Weight (kg)	3464	66.8±10.4	703	55.8±6.0	671	62.3±4.2	680	65.7±4.4	671	69.6±5.1	739	79.7±10.1
Height (m)	3464	1.74±0.06	703	1.74±0.06	671	1.74±0.06	680	1.74±0.06	671	1.74±0.06	739	1.75±0.06
**Measurements at mean age** **50 years (middle-age)**												
Age (years)	3464	48.8±5.5	703	49.6±5.7	671	49.0±5.3	680	48.8±5.4	671	48.3±5.3	739	48.2±5.5
BMI (kg/m^2^)	3464	25.4±3.0	703	23.7±2.9	671	24.4±2.5	680	25.1±2.4	671	25.9±2.5	739	27.6±3.0
normal-weight (BMI<25 kg/m^2^)	1628	47.0%										
overweight (25 kg/m^2^≤BMI<30 kg/m^2^)	1606	46.4%										
obese (BMI≥30 kg/m^2^)	230	6.6%										
Weight (kg)	3464	77.0±10.3	703	71.8±9.7	671	73.9±8.7	680	75.9±8.6	671	78.5±9.1	739	84.2±10.4
Height (m)	3464	1.74±0.06	703	1.74±0.07	671	1.74±0.06	680	1.74±0.06	671	1.74±0.07	739	1.74±0.07
**Measurements at mean age** **70 years (late adulthood)**												
Age (years)	3464	68.6±5.5	703	69.4±5.7	671	68.7±5.3	680	68.6±5.4	671	68.1±5.3	739	68.0±5.5
BMI (kg/m^2^)	3464	27.0±3.7	703	25.3±3.6	671	26.1±3.3	680	26.5±3.2	671	27.4±3.2	739	29.3±3.9
normal-weight (BMI<25 kg/m^2^)	1054	30.4%										
overweight (25 kg/m^2^≤BMI<30 kg/m^2^)	1799	51.9%										
obese (BMI≥30 kg/m^2^)	611	17.6%										
Weight (kg)	3464	80.3±12.5	703	74.9±11.6	671	77.5±11.0	680	78.8±10.9	671	81.8±11.5	739	87.9±13.1
Height (m)	3464	1.72±0.06	703	1.72±0.07	671	1.72±0.06	680	1.72±0.06	671	1.73±0.07	739	1.73±0.06
Total cholesterol (mmol/L)	3287	6.0±1.1	663	6.0±1.1	635	6.1±1.1	642	6.0±1.0	642	6.0±1.1	705	6.0±1.1
LDL cholesterol (mmol/L)	3244	3.9±1.0	654	3.8±1.0	627	4.0±1.0	631	3.9±0.9	637	3.9±1.0	695	3.9±1.0
SBP (mmHg)	3450	148.8±24.2	702	148.6±25.1	667	149.1±24.2	679	148.4±24.1	665	148.8±23.6	737	148.8±24.2
DBP (mmHg)	3450	85.1±11.1	702	84.6±11.6	667	84.8±10.8	679	84.6±11.1	665	85.9±11.0	737	85.4±11.1
Insulin (µΙU/mL)^a^	3280	8.7 (5.7, 12.4)	666	8.6 (5.5, 12.6)	632	8.5 (5.6, 12.3)	642	8.3 (5.5, 11.8)	641	8.3 (5.6, 11.6)	699	9.6 (6.4, 13.7)
Glucose (mmol/L)^a^	3287	5.8 (5.2, 6.1)	664	5.8 (5.3, 6.0)	636	5.8 (5.2, 6.1)	641	5.8 (5.2, 6.1)	643	5.8 (5.2, 6.0)	703	5.9 (5.2, 6.2)
HbA1c (%)	3287	5.0±0.9	666	5.0±0.9	635	5.0±0.8	642	5.0±0.8	643	5.0±0.9	701	5.1±1.0
HbA1c (mmol/mol)	3280	31.3±9.8	665	31.4±9.5	634	30.8±9.0	641	30.8±9.0	641	31.3±10.2	699	32.3±11.0

n: Number of participants (subset of individuals with BMI available at all age periods).

SD: Standard deviation.

a: Estimates correspond to geometric mean (Q1, Q3).

Quintiles of BMI at 21 years are calculated using all available data (including individuals with missing BMI at mean age 50 or 70 years).

Table 2 shows the difference in cardiovascular and metabolic risk markers at 70 years of age, associated with a 1 kg/m^2^ higher BMI at each different age-group (BMI-21, BMI-50, BMI-70) separately. BMI at all age periods was significantly associated with insulin, glucose and HbA1c. However, these associations were weaker for BMI-21 than for BMI-50 and BMI-70 (regression coefficients for BMI-21 were a fifth for serum insulin, and between a half to a third for blood glucose and HbA1c, of those for BMI-50 and BMI-70). BMI-50 and BMI-70 showed positive associations with systolic and diastolic blood pressure, while BMI-21 showed no association. LDL-cholesterol showed no association with BMI at any age; total cholesterol was only associated with BMI-70. There was no evidence to suggest that the associations between BMI-70 and any risk marker were modified by the level of BMI-21 (i.e., there was no interaction between BMI-70 and quintiles of BMI-21 - Table S1 in File S1).

**Table 2:** Differences in cardiovascular and diabetes risk markers at mean age 70 years for each 1 kg/m^2^ increase in BMI separately at mean age 21, 50 and 70 years.

Outcome (mean age 70 years)	BMI at mean age 21 years			BMI at mean age 50 years			BMI at mean age 70 years		
	n	Coef (95% CI)	p-value	n	Coef (95% CI)	p-value	n	Coef (95% CI)	p-value
Total cholesterol (mmol/L)	3287	–0.009 (–0.022, 0.003)	0.15	3287	–0.007 (–0.020, 0.005)	0.260	3287	0.011 (0.001, 0.021)	0.03
LDL cholesterol (mmol/L)	3244	–0.007 (–0.018, 0.005)	0.25	3244	–0.009 (–0.020, 0.0022)	0.115	3244	0.005 (–0.005, 0.014)	0.32
SBP (mmHg)	3450	0.123 (–0.149, 0.395)	0.37	3450	0.492 (0.223, 0.761)	<0.001	3450	0.696 (0.481, 0.910)	<0.001
DBP (mmHg)	3450	0.083 (–0.043, 0.210)	0.20	3450	0.192 (0.067, 0.318)	0.003	3450	0.336 (0.236, 0.436)	<0.001
Insulin (% difference)	3280	1.525 (0.771, 2.285)	<0.001	3280	7.614 (6.862, 8.371)	<0.001	3280	8.587 (8.014, 9.164)	<0.001
Glucose (% difference)	3287	0.362 (0.127, 0.598)	0.003	3287	1.096 (0.863, 1.330)	<0.001	3287	0.733 (0.545, 0.922)	<0.001
HbA1c (%)	3287	0.016 (0.006, 0.026)	0.002	3287	0.046 (0.036, 0.056)	<0.001	3287	0.030 (0.022, 0.038)	<0.001
HbA1c (mmol/mol)	3280	0.177 (0.067, 0.288)	0.002	3280	0.488 (0.380, 0.597)	<0.001	3280	0.322 (0.234, 0.410)	<0.001

n: Number of participants (subset of individuals with BMI available at all age periods).

Coef: regression coefficient represent difference in cardiovascular risk markers for 1 kg/m2 increase in BMI. Estimates are adjusted for age at the time when the risk markers were measured, and town as a fixed effect.

Table 3 shows the difference in cardiovascular and diabetes risk markers at 70 years of age associated with having high BMI (a BMI on or above the 75th centile) separately at mean age 21, 50 and 70 years, when compared to men who never had high BMI (separate analyses based on sensitive period models). Having high BMI at any one of these three ages was associated with raised levels of insulin, glucose, HbA1c and diastolic blood pressure at mean age 70 years, though the differences were greater for high BMI-50 and BMI-70 than for BMI-21. Additionally, the presence of a high BMI at mean age 50 and 70 years was associated with higher systolic pressure at mean age 70 years. Over 40% of individuals with high BMI at mean age 21 and over 70% of individuals with high BMI at mean age 50 years also had high BMI at mean age 70 years. Hence, the effects of high BMI at different periods were not independent.

**Table 3:** Differences in cardiovascular and diabetes risk markers at mean age 70 years, associated with having high BMI (≥75^th^ centile) separately at mean ages 21, 50 and 70 years compared to men with normal BMI at all 3 ages (separate analyses based on sensitive period models).

	Sensitive period											
Outcome (mean age 70 years)	Never had high BMI			High BMI at mean age 21 years			High BMI at mean age 50 years			High BMI at mean age 70 years		
	n	K	Coef (95% CI)	n	K	Coef (95% CI)	n	K	Coef (95% CI)	n	K	Coef (95% CI)
												
Total cholesterol (mmol/L)	1898	0%	reference	868	44.12%	–0.07 (–0.16, 0.02)	717	70.57%	–0.07 (–0.16, 0.03)	802	100%	–0.01 (–0.10, 0.08)
LDL cholesterol (mmol/L)	1880	0%	reference	858	43.94%	–0.08 (–0.15, 0.00)	698	70.2%	–0.10 (–0.18, −0.01)	783	100%	–0.06 (–0.14, 0.02)
SBP (mmHg)	1981	0%	reference	909	46.09%	1.82 (–0.06, 3.70)	768	71.61%	3.18 (1.17, 5.18)	871	100%	4.56 (2.64, 6.47)
DBP (mmHg)	1981	0%	reference	909	46.09%	0.99 (0.11, 1.87)	768	71.61%	1.63 (0.70, 2.57)	871	100%	2.03 (1.14, 2.92)
Insulin (% difference)	1896	0%	reference	862	44.43%	24.45 (18.42, 30.78)	712	70.93%	55.04 (47.01, 63.51)	803	100%	68.66 (60.49, 77.26)
Glucose (% difference)	1898	0%	reference	869	44.07%	3.02 (1.37, 4.69)	713	70.41%	7.35 (5.52, 9.21)	801	100%	5.68 (3.94, 7.44)
HbA1c (%)	1901	0%	reference	866	44.46%	0.16 (0.09, 0.23)	713	70.97%	0.33 (0.26, 0.40)	804	100%	0.27 (0.20, 0.34)
HbA1c (mmol/mol)	1896	100%	reference	864	44.44%	1.76 (1.00, 2.51)	712	70.93%	3.58 (2.77, 4.39)	803	100%	2.92 (2.15, 3.70)

n: Number of participants (subset of individuals with BMI available at all age periods).

K: percentage of individuals who had high BMI (BMI in the top 25% of the distribution) also at mean age 70 years.

Coef: regression coefficient for the effect of high BMI (BMI in the top 25% of the distribution) at each sensitive period (mean age 21, 50 or 70 years) as compared with never having had high BMI. Estimates are adjusted for age at the time when the risk markers were measured, and town as fixed effect.

For each outcome, results are obtained from 3 models fitted separately (one for each period: mean age 21, 50 and 70 years).

Table 4 shows the differences in cardiovascular and diabetes risk markers at 70 years of age associated with each life course BMI trajectory (having a high BMI at different points of the life course), expressed as the difference in risk markers from the reference (0–0–0) trajectory (i.e., where high BMI was not present at any age). The strongest effects were apparent for insulin. Having a high BMI in at least one age period, when compared to normal BMI at all ages, was associated with higher insulin levels at mean age 70 years, with the exception of those with high BMI only at age 21 years. High BMI-70 was associated with the highest levels of insulin. Glucose and HbA1c were both higher when BMI-50 was higher; HbA1c alone was increased when BMI-70 was increased. Men with both high BMI-50 and BMI-70 showed higher levels of systolic and diastolic blood pressure when compared to men who never had high BMI; men with high BMI-70 only also had higher systolic blood pressure. Men with a high BMI at age 21 years and normal BMI at 50 and/or 70 years tended to have similar blood pressures at 70 years to men who had never had high BMI. There was an exception to this pattern; men with high BMI at 21 and 70 years but not at age 50 years had increased levels of diastolic blood pressure when compared with those who never had high BMI. Total and LDL cholesterol levels were not appreciably associated with BMI at any age.

**Table 4:** Differences in cardiovascular and diabetes risk markers at mean age 70 years, by trajectory of BMI (having a high BMI at different points of the life course) at different ages (mean age 21, 50 and 70 years).

BMI at mean age 70 years (kg/m^2^)			Total cholesterol		LDL cholesterol		SBP		DBP		Insulin		Glucose		HbA1c		HbA1c	
			(mmol/L)		(mmHg)		(mmHg)		(mmHg)		(% difference)		(% difference)		(%)		(mmol/mol)	
BMI trajectory	n	mean (SD)	n	Coef (95% CI)	n	Coef (95% CI)	n	Coef (95% CI)	n	Coef (95% CI)	n	Coef (95% CI)	n	Coef (95% CI)	n	Coef (95% CI)	n	Coef (95% CI)
0–0-0	1988	25.0 (2.3)	1898	0 (ref. group)	1880	0 (ref. group)	1981	0 (ref. group)	1981	0 (ref. group)	1896	0 (ref. group)	1898	0 (ref. group)	1901	0 (ref. group)	1896	0 (ref. group)
1–0-0	383	26.1 (2.0)	376	–0.07 (–0.19, 0.05)	373	–0.05 (–0.15, 0.06)	380	0.84 (–1.78, 3.45)	380	0.35 (–0.87, 1.57)	374	–4.68 (–10.77, 1.83)	377	–0.410 (–2.57, 1.79)	375	0.03 (–0.06, 0.13)	374	0.36 (–0.67, 1.39)
0–1-0	109	27.2 (1.6)	102	–0.13 (–0.34, 0.08)	100	–0.16 (–0.35, 0.04)	108	0.34 (–4.29, 4.97)	108	1.36 (–0.79, 3.52)	102	21.28 (7.67, 36.60)	102	6.75 (2.61, 11.06)	101	0.35 (0.18, 0.52)	101	3.73 (1.86, 5.59)
1–1-0	111	27.5 (1.2)	109	–0.20 (–0.41, 0.002)	108	–0.17 (–0.35, 0.02)	110	–0.67 (–5.25, 3.90)	110	–0.43 (–2.56, 1.70)	105	18.34 (5.27, 33.02)	109	4.52 (0.60, 8.60)	106	0.21 (0.04, 0.38)	106	2.25 (0.43, 4.06)
0–0-1	219	30.8 (1.9)	202	0.00 (–0.15, 0.16)	201	–0.05 (–0.19, 0.09)	218	5.03 (1.69, 8.37)	218	1.49 (–0.06, 3.05)	203	69.01 (55.04, 84.24)	202	2.27 (–0.63, 5.26)	201	0.16 (0.03, 0.28)	201	1.64 (0.28, 2.99)
1–0-1	103	30.7 (2.0)	94	0.06 (–0.16, 0.28)	92	–0.05 (–0.25, 0.15)	103	3.73 (–1.01, 8.47)	103	2.78 (0.57, 4.99)	95	48.90 (31.63, 68.43)	97	0.60 (–3.40, 4.77)	97	0.09 (–0.09, 0.26)	97	0.85 (–1.05, 2.76)
0–1-1	235	32.0 (2.8)	217	0.019 (–0.13, 0.17)	205	–0.04 (–0.18, 0.10)	234	6.12 (2.89, 9.36)	234	2.63 (1.12, 4.14)	217	74.94 (60.86, 90.24)	216	8.12 (5.14, 11.18)	218	0.37 (0.25, 0.49)	218	3.96 (2.65, 5.26)
1–1-1	316	32.8 (3.0)	289	–0.05 (–0.19, 0.08)	285	–0.09 (–0.21, 0.03)	316	3.32 (0.48, 6.16)	316	1.71 (0.38, 3.03)	288	70.58 (58.39, 83.70)	286	8.09 (5.44, 10.79)	288	0.34 (0.23, 0.44)	287	3.73 (2.57, 4.89)

n: Number of participants (subset of individuals with BMI available at all age periods). For BMI at mean 70 years this includes all available data.

BMI trajectories: Each triplet corresponds to a different trajectory of high BMI at mean age 21, 50 and 70 years; with 0 and 1 denoting BMI below and above the 75th percentile of the BMI distribution, respectively. For example, (0–0-0) denoted low BMI at all age periods, whilst (0–0-1) signified high BMI at mean age 70 years only.

Coef: Estimates are differences in risk marker from BMI trajectory 0–0-0. Estimates are adjusted for age at the time when the risk markers were measured, and town as fixed effect.

Compared with the saturated model (8 BMI trajectories presented in table 4) the likelihood ratio tests (LRT) suggested a sensitive period to high BMI-50 years for glucose and HbA1c at 70 years (LRT p = 0.49 and p = 0.16 respectively) and a sensitive period to high BMI-70 years for blood pressure at 70 years (LRT p = 0.86 for systolic blood pressure and p = 0.69 for diastolic blood pressure). For insulin however, none of the other life course models (sensitive period, accumulation or mobility) fitted the data as well as the saturated model.

Additional adjustment for smoking in all models made little difference to the results. The exclusion of participants on lipid lowering medication, blood pressure lowering medication or blood glucose lowering medication did not affect the results presented in Tables 2–4 for total and LDL cholesterol, blood pressure and glucose, insulin, HbA1c respectively (data not presented).

## Discussion

Although adiposity in middle-age is an established risk factor for cardiovascular disease and type 2 diabetes,[5]–[8] the impact of high BMI from early adult life on these outcomes is not clearly established. Birth cohort studies often have information on adiposity both before and during middle-age,[18]–[20] but generally have populations which are too young to have large numbers of chronic disease events in later life. Many prospective studies have data on adiposity in middle-age and its relation to subsequent chronic disease outcomes [5], [28] but few of these also have information on adiposity in early adult life.[16] This study provides novel information on the association between high BMI at different points of the life course and cardiovascular and metabolic risk factors in later life. Findings are based on a large white European male cohort born in the early 20th century, who were thin compared to more contemporary cohorts. While early BMI had little influence on cardiovascular risk factors, it was associated with metabolic factors related to type 2 diabetes risk, suggesting some early patterning of diabetes risk. This may have greater relevance to more recent cohorts with higher levels of BMI from an early age.

### Previous studies

Our observation that BMI in later life was a considerably more important determinant of both cardiovascular and metabolic risk than BMI in early life is in agreement with an overwhelming body of evidence showing that adiposity and weight gain in middle-age show strong, graded associations with cardiovascular disease, type 2 diabetes and their precursors in later life.[5]–[8], [15] In the present study, early BMI had a modest association with metabolic markers related to diabetes risk, though not with cardiovascular risk factors. Further support of an association between early life BMI and diabetes risk is evident from our life course approach; for serum insulin none simpler model (sensitive period, mobility or accumulation models) fitted the data as well as the saturated model. This is consistent with the results of recent studies which have suggested that adiposity in early adult life is strongly related to the early development of T2D,[10], [29] and that a greater duration of adiposity may have an important impact on T2D risk.[11] This is also coherent with the observation in Israeli army recruits that high BMI in adolescence was related to type 2 diabetes in later life; in that study the association was abolished after adjustment for adult BMI.[30] That study also reported that weight loss in early adult life among overweight adolescents may be associated with lower diabetes risk.[30] In the present study, high BMI in early adult life followed by normal BMI in middle and later life was associated with similar or marginally lower insulin and glucose levels in later life, again suggesting that the effects of high BMI in early life may be reversible by subsequent weight loss.

The present study found no evidence that early BMI related to blood pressure and blood cholesterol levels. Previous analyses examining the associations of BMI at different ages on blood pressure and blood cholesterol at 45 years in the 1958 British birth cohort (NCDS)[12], [31] were consistent with the present study in observing that current BMI had much stronger associations than earlier BMI with blood pressure and non-HDL cholesterol. However, in contrast with our findings, BMI at 23 years showed weak positive associations with both blood pressure and non-HDL cholesterol at 45 years. In the 1958 British birth cohort, the associations between current BMI and both blood pressure and non-HDL cholesterol were modified by earlier BMI, being particularly strong in individuals who were thinner at younger ages; we were unable to confirm these findings in the BRHS. Both the present study and the 1958 British birth cohort were however consistent in showing that recent weight gain was most strongly associated with blood pressure level in middle or later life;[12] in the 1958 British birth cohort but not in the present study, similar associations were observed for non-HDL cholesterol.[31] Taken together, the limited evidence relating early adult BMI to later cardiovascular risk in the present study is consistent with the conclusions of a recent review.[32].

### Strengths and limitations

The investigation is based on a geographically and socially representative cohort study with high response rates and exceptionally high follow-up rates. This study has used a novel approach in extending an established cohort of middle aged men by using data on BMI from military service records as a source of information in early adulthood. Among men who survived to the 60–79 year re-examination, it was possible using the combination of two sources of information, military health records and participant recall, to obtain information on BMI at 21 years for a high proportion of participants (82%); information from military records was documented at the time of measurement and recalled data were substantially consistent. The availability of data from both sources in 694 men allowed adjustment for a small bias in recalled weight and validated the use of recalled weight. Although the analyses were based on slightly fewer than half of participants in the original cohort, inevitably being based on a healthy survivor group, baseline risk factor differences in men who were or were not included in the present analyses did not differ markedly (data not presented). Hence, underestimation of potential associations between early BMI and later cardiometabolic risk because of earlier mortality amongst participants at greatest risk is likely to be limited. A further concern is the extended time interval between BMI at 21 years and cardiometabolic outcomes about 50 years later, by which point the development of disease could make the detection of associations difficult. However, the results for blood lipids, blood pressure and insulin and glycaemia markers were not materially affected by exclusion of study participants receiving medications likely to have affected these outcome measures. BMI is only one of many surrogate markers of adiposity. Unfortunately, other measures of adiposity such as waist circumference, waist to hip ratio and percentage of fat mass were only available for the 60–79 year examination and their use would have undermined a key strength of the current analysis, that the same marker of adiposity was used for all age groups. Moreover, BMI is highly correlated with fat mass both in this population (at 70 years, r = 0.70, p<0.001). Although BMI is a robust measure of overall adiposity, its association with body fatness differs with age; in later life changes in muscle mass as well as fatness are important influences on BMI.[33] Reassuringly, the exclusion of men aged 75 years and over (in whom loss of muscle mass is likely to influence BMI [34]) made little difference to the findings. However, other direct measures of adiposity (including central adiposity) could help to explain why associations between adiposity and cardiovascular risk markers were limited in strength or absent. In addition, the limited number of study participants with high levels of adiposity in early life limits the ability of the investigation to detect the effects of adiposity at this stage of the life course. The analytic strategies used in this report focus on the comparative strength and consistency of independent associations between adiposity at each age time point (in early adulthood, middle-age, and later life) and cardio-metabolic risk in later life, corresponding with models used in other relevant recent reports.[12], [35], [36] We have avoided models using mutual adjustment for BMI at different stages of the life course, which have been shown to yield confusing or erroneous estimates of associations [37], [38] (Statistical appendix S1 in File S1). Moreover, we have assessed how different life course patterns and potentially cumulative or combined associations between BMI at different stages of the life course are associated with cardio-metabolic risk, comparing the presence of high BMI at three, two, one and no stages of the life course.

### Implications

The results suggest that BMI in later life is the dominant influence on risk markers both for CHD and for type 2 diabetes. Early adult BMI level may be associated with later type 2 diabetes risk, but not with markers of CHD risk. However, it is not possible to establish whether this association reflects the positive correlation between BMI in early adult life and BMI in later life, or whether it reflects a more direct association between early BMI and diabetes risk, possibly based on the cumulative effects of BMI on insulin resistance and pancreatic beta cell function over an extended period from early adult life. This uncertainty partly reflects the limitations of the present cohort for investigating this issue, given the low levels of adiposity and the low prevalence of overweight/obesity in early adult life observed in the present cohort. However, the associations between adiposity, diabetes and CHD in the present cohort (with a lesser burden of adiposity in early adult life than that in subsequent cohorts) could act as a valuable reference point for investigating the health effects of adiposity in subsequent birth cohorts, in which adiposity in early adult life became more marked. While findings from this study are generalizable to elderly white males, relevance to women of a similar age (who show different patterns and trends in body composition, e.g. greater losses in muscle mass with age), and other ethnic groups remains to be established. This work will be carried on when cohort studies with individuals with higher levels of adiposity in early life will become available.

## Supporting Information

File S1 **Supplemental material.** Figure S1, Association between weight at 21 years recalled in 1996 and weight measured during military service at age closest to 21 years (and between 20 and 22 years). Table S1, Regression coefficients showing the associations between BMI at 70 years (per 1 kg/m increase) and cardiovascular and diabetes risk factors at mean age 70 years, stratified by quintiles of BMI at 21 years. Statistical appendix S1, Justification for analytic approach. Data S1, Data underlying the findings described in the manuscript.
(ZIP)
