# Supplementary material for: Adiposity in Early, Middle and Later Adult Life and Cardiometabolic Risk Markers in Later Life; Findings from the British Regional Heart Study
Source: PLoS One. 2014 Dec 4;9(12):e114289. doi: 10.1371/journal.pone.0114289 (PMC4256406; doi:10.1371/journal.pone.0114289)
Supplement: File S1 — Supplemental material. Figure S1, Association between weight at 21 years recalled in 1996 and weight measured during military service at age closest to 21 years (and between 20 and 22 years). Table S1, Regression coefficients showing the associations between BMI at 70 years (per 1 kg/m increase) and cardiovascular and diabetes risk factors at mean age 70 years, stratified by quintiles of BMI at 21 years. Statistical appendix S1, Justification for analytic approach. Data S1, Data underlying the findings described in the manuscript. (ZIP) [file pone.0114289.s001.zip › Statistical appendix S1.docx]

# Statistical appendix S1: Justification for analytic approach

In the present study, there was no association between BMI in early adulthood and cardiovascular risk factors, and the associations between early BMI and metabolic risk markers in late adulthood became either null or showed a statistically significant inverse association after adjustment for BMI in later life. We have not presented these data as adiposity levels at different age periods are likely to be strongly correlated and analyses which include mutual adjustment for adiposity measures at different stages of the life course are likely to be misleading.[1, 2] For instance, the reversal of associations after adjustment demonstrates the so called “reversal paradox”.[1, 3] This can either be interpreted as a statistical artefact or a potentially a protective effect of elevated BMI in early life.[1, 3]

Statistical analyses that adjust for longitudinal exposure variables by including all measurements in the model can be parameterised in more than one way (e.g. including exposure measurements at each stage, or initial measurement and consequent changes). The interpretation of the regression coefficients from such models depends on the chosen parameterisation.[4, 5] This may result in erroneous conclusions. For exposure variables that are irreversible (e.g. height is an increasing function of age) including initial measurement and consequent changes may be biologically meaningful.[6] However, for exposure variables that may either increase or decrease over time (e.g. BMI), any parameterisation will be subject to having cross-sectional associations being intertwined with longitudinal changes. Including the whole BMI trajectory using a life course approach when investigating the effect of different BMI patterns to an outcome rather than using parts of the causal pathway as confounders avoids these caveats.

We will illustrate the potential confusion arising from such models in the context of diastolic blood pressure (DBP). In table 2, BMI at 21 does not appear to be related to DBP (0.083mmHg per 1 kg/m² rise in BMI at 21, p=0.20). If we additionally adjust for BMI at 70 years (Model 1), the coefficient for BMI at 21 years is reversed (-0.087mmHg per 1 kg/m² rise in BMI, p=0.21); the coefficient for BMI at 70 years remains about the same (0.362 mmHg per 1 kg/m² rise in BMI at 70, p<0.001).

However, by adjusting for BMI change from 21 to 70 years instead of BMI at 70 years (Model 2) the coefficient for BMI at 21 becomes stronger and statistically significant 0.275 mmHg per 1 kg/m² rise in, (p<0.001) and for BMI change between 21 and 70 years it is 0.362 per mmHg per 1 kg/m² rise in (p<0.001). Alternatively we could adjust for BMI 70 and BMI change from 21 to 70 years (Model 3) in which case the coefficients are respectively 0.275 mmHg per 1 kg/m² rise, (p<0.001) and 0.087 per mmHg per 1 kg/m² rise (p=0.21). Thus, depending on what we chose to condition our model on, the independent effect of BMI at 21 years on diastolic blood pressure can be either null or positive and statistically significant. Some coefficients appear to be of the same magnitude, and others appear to be the sum of other coefficients e.g. 0.362 – 0.087 = 0.275; this is summarized below.

| **Model adjusts for:** | **BMI 21yrs** | **BMI 70yrs** | **BMI change from 21 to 70 yrs** |
| --- | --- | --- | --- |
| Model 1 | -0.087 (p=0.21) | 0.362 (p<0.001) |  |
| Model 2 | 0.275 (p<0.001) |  | 0.362 (p<0.001) |
| Model 3 |  | 0.275 (p<0.001) | 0.087 (p=0.21) |

The reason why certain coefficients appear of the same magnitude is because the three parameterizations are algebraically identical since they describe the same underlying model. For exposure variables such as height, where a 1cm increase in height in childhood has an irreversible contribution to adult height (excluding shrinkage in later life), including initial measurement and conditioning on subsequent changes may be biologically meaningful.[6] However, this approach may often lead to confusion and misinterpretation of the results especially when exposure variables can both increase and decrease over time. The life course approach that we have followed avoids this complication by modelling explicitly every possible trajectory of high BMI over the life course.

# References

(1) Tu YK, West R, Ellison GT, Gilthorpe MS. (2005) Why evidence for the fetal origins of adult disease might be a statistical artifact: the "reversal paradox" for the relation between birth weight and blood pressure in later life. Am J Epidemiol 161(1):27-32.

(2) Park MH, Falconer C, Viner RM, Kinra S. (2012) The impact of childhood obesity on morbidity and mortality in adulthood: a systematic review. Obes Rev 13(11):985-1000.

(3) Lloyd LJ, Langley-Evans SC, McMullen S. (2009) Childhood obesity and adult cardiovascular disease risk: a systematic review. Int J Obes (Lond).

(4) Lucas A, Fewtrell MS, Cole TJ. (1999) Fetal origins of adult disease-the hypothesis revisited. BMJ 319(7204):245-9.

(5) De Stavola BL, Nitsch D, dos Santos Silva I, McCormack V, Hardy R, et al. (2006) Statistical issues in life course epidemiology. Am J Epidemiol 163(1):84-96.

(6) Cole TJ, Fewtrell M, Lucas A. (2001) Early growth and coronary heart disease in later life - Analysis was flawed. British Medical Journal 323(7312):572-3.
