# Supplementary figures and images for: Adiposity in Early, Middle and Later Adult Life and Cardiometabolic Risk Markers in Later Life; Findings from the British Regional Heart Study
Source: PLoS One. 2014 Dec 4;9(12):e114289. doi: 10.1371/journal.pone.0114289 (PMC4256406; doi:10.1371/journal.pone.0114289)

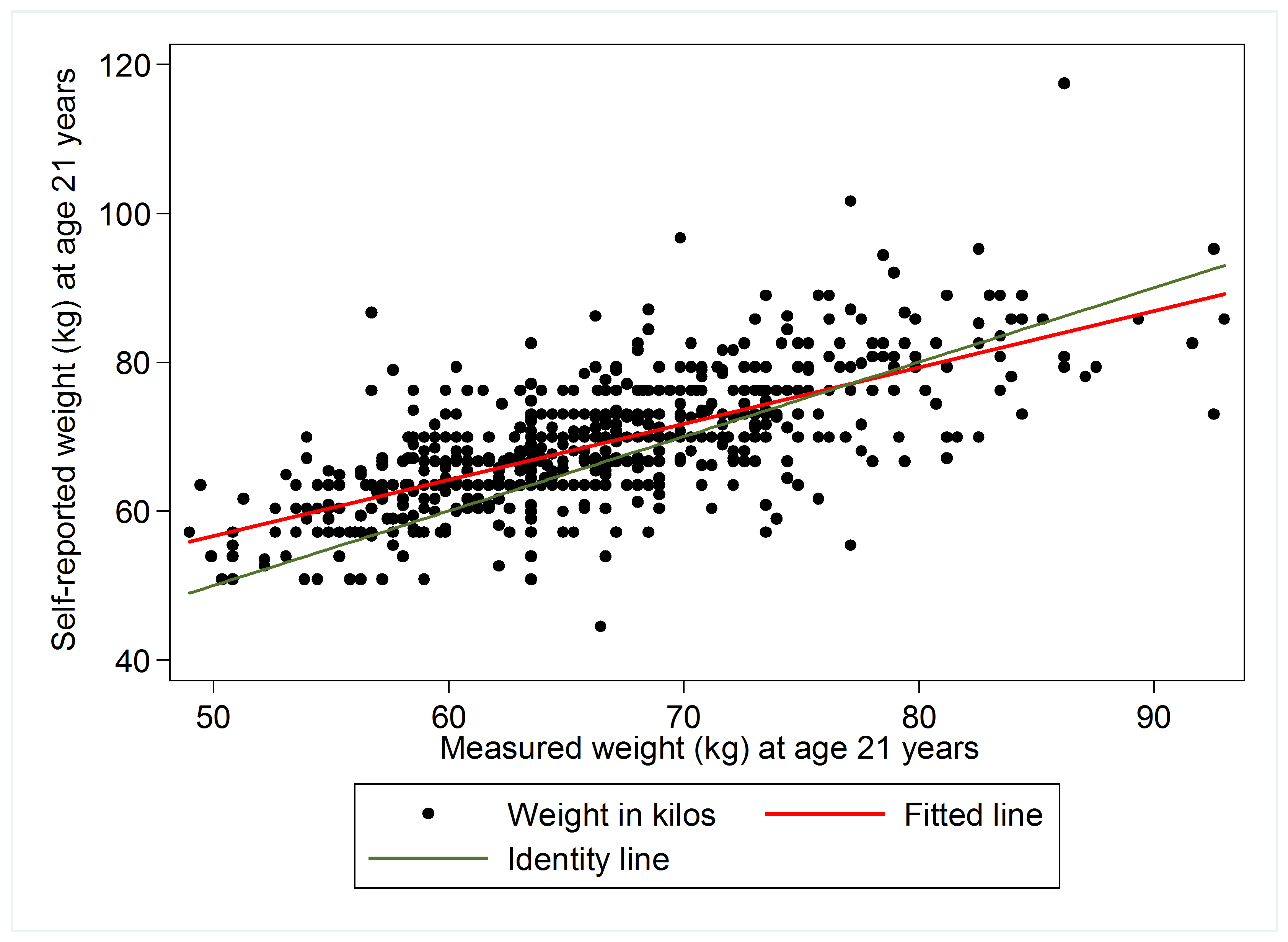

Supplement: File S1 — Supplemental material. Figure S1, Association between weight at 21 years recalled in 1996 and weight measured during military service at age closest to 21 years (and between 20 and 22 years). Table S1, Regression coefficients showing the associations between BMI at 70 years (per 1 kg/m increase) and cardiovascular and diabetes risk factors at mean age 70 years, stratified by quintiles of BMI at 21 years. Statistical appendix S1, Justification for analytic approach. Data S1, Data underlying the findings described in the manuscript. (ZIP) [file pone.0114289.s001.zip › Figure S1.tiff]
